# Can Sleep and Resting Behaviours Be Used as Indicators of Welfare in Shelter Dogs (*Canis lupus familiaris*)?

**DOI:** 10.1371/journal.pone.0163620

**Published:** 2016-10-12

**Authors:** Sara C. Owczarczak-Garstecka, Oliver H. P. Burman

**Affiliations:** 1 Institute for Risk and Uncertainty, University of Liverpool, Liverpool, United Kingdom; 2 School of Life Science, University of Lincoln, Lincoln, United Kingdom; University of Sydney Faculty of Veterinary Science, AUSTRALIA

## Abstract

Previous research on humans and animals suggests that the analysis of sleep patterns may reliably inform us about welfare status, but little research of this kind has been carried out for non-human animals in an applied context. This study explored the use of sleep and resting behaviour as indicators of welfare by describing the activity patterns of dogs (*Canis lupus familiaris*) housed in rescue shelters, and comparing their sleep patterns to other behavioural and cognitive measures of welfare. Sleep and activity patterns were observed over five non-consecutive days in a population of 15 dogs. Subsequently, the characteristics of sleep and resting behaviour were described and the impact of activity on patterns of sleep and resting behaviour analysed. Shelter dogs slept for 2.8% of the day, 14.3% less than previously reported and experienced less sleep fragmentation at night (32 sleep bouts). There were no statistically significant relationships between behaviours exhibited during the day and sleep behaviour. A higher proportion of daytime resting behaviour was significantly associated with a positive judgement bias, less repetitive behaviour and increased time spent coded as ‘relaxed’ across days by shelter staff. These results suggest that, in the context of a busy shelter environment, the ability to rest more during the day could be a sign of improved welfare. Considering the non-linear relationship between sleep and welfare in humans, the relationship between sleep and behavioural indicators of welfare, including judgement bias, in shelter dogs may be more complex than this study could detect.

## Introduction

Over 130,000 dogs (*Canis lupus familiaris*) enter animal shelters in the UK each year and nearly one third of the UK’s 10 million dog population have been in a shelter at some point [1]. Exposure to unfamiliar surroundings or being separated from familiar people can be stressful for dogs and contribute to the experience of poor welfare [2]. Therefore, the ability to assess dog welfare is important to design appropriate interventions. Common welfare indicators include physiological measures (e.g. cortisol fluctuation), behavioural measures (e.g. the presence of behaviours associated with an increasing stress response, like panting) and cognitive measures (e.g. judgement bias test) [3], all of which can vary in their efficacy and levels of invasiveness [4]. A little researched indicator is sleep behaviour (e.g. [5]), defined as “a state of immobility with greatly reduced responsiveness” [6] (p. 1264). Sleep has been identified as a promising indicator of welfare because it can be used as an index of adaptation to the environment [7, 8].

The sleep cycle of most mammals, including dogs, consists of slow-wave sleep or quiet sleep, followed by REM sleep (Rapid Eye Movement sleep, also called active sleep), and then wakefulness [9]. Changes in sleep architecture, that is changes in the order, latency to onset and duration of individual phases of the sleep cycle, occur in response to events experienced when awake [9]. For example, mild *chronic* stress in the form of unpredictable husbandry procedures has been found to decrease latency to the first REM sleep bout, increase absolute time of REM sleep, increase the number of behavioural transitions during the REM sleep phase, and decrease sensitivity to rewards in rats [10] indicative of negative welfare. Moreover, others have found that *acute* stress (e.g. an immobilization test) in rats results in an increase of REM sleep bouts during the sleep and wake phases compared to baseline, an increase in quiet sleep in those individuals who during baseline conditions slept least, and a decrease in quiet sleep in those individuals who, during baseline conditions, slept more than average [11]. This implies that individual coping styles may be reflected in sleep changes following experience of a stressful event.

Importantly, lack of sleep is a major stressor in itself. Reducing the amount of sleep substantially below average (e.g. see Banks & Dinges [12]) is linked with psychomotor and sensorimotor deterioration, disinhibition of responses to negative stimuli, anxiety, aggression, anhedonia (defined as a decrease in sensitivity to rewards [13]), lower frustration tolerance [14], as well as a poorer ability to cope with stressful stimuli [15]. Humans suffering from depression also experience changes in their sleep architecture, and research indicates higher than expected comorbidity between sleep disorders and depression [16]. This suggests that disturbances to normal sleep routines and sleep deprivation may also have a negative impact on animal welfare. Indeed, in both rats and humans, sleep disturbances elevate hypothalamic-pituitary-adrenal axis activity during the day, leading to changes in neuroendocrine functioning (e.g. alterations in corticotrophin-releasing factor in the long term, or elevation of cortisol and adrenal corticotropic hormone levels in the short term), and strongly attenuated adrenal corticotropic hormone responses to stressors, compared to subjects who get sufficient sleep [17, 18]. Therefore, subjects who do not get enough sleep are less able to cope with stressful stimuli [17, 19]. In laboratory rats, the frequency of quiet sleep bouts and the total duration of sleep have shown to negatively correlate with adrenal weight and positively correlate with bodyweight gain and final bodyweight [20]. Together, this suggests that quiet sleep and REM sleep change in response to stress. Moreover, rats that experienced disturbance of sleep by husbandry routines showed decreased self-grooming and enrichment-directed behaviours (defined as sniffing, chewing, climbing, and manipulating enrichment objects), lighter thymus glands and higher aggression scores [20], indicating an effect of sleep disturbance on commonly used physiological indicators of welfare.

Despite ample evidence that sleep has a significant relationship with mental and physical wellbeing, to date, few studies have used sleep as an indicator of welfare in animals in an applied context. Instead, most studies have focused on states of inactivity more generally. For example, increased inactivity while awake reflects compromised welfare in cats [21], rodents [22] and mink [23]. However, inactivity is also linked with relaxation and may reflect increased comfort in the environment [24, 25]. The difference between inactivity caused by distress and caused by relaxation is evident in the position of resting [23]. For example, distress may induce positions that facilitate rapid mobilization, location of resting (e.g. in view or hiding; [23]) and fragmentation of resting bouts, where distressed animals are likely to prioritise scanning the environment and, thus, experience shorter sleeping and resting bouts [26].

Dogs follow a diurnal circadian rhythm [27] but the percentage of time spent asleep depends on the population of dogs studied. Unrestricted dogs slept during 60% of the night, whilst dogs entering a barren research facility slept for over 80% of the night [27] and dogs in a modern research facility, where environmental enrichment was provided, slept between 60–71% of the night and 30–37% of the day [28]. Other factors that appear to influence sleep in dogs include diet and frequency of feeding [29], changes in housing conditions [27], changes in working routine [30] and activity levels during the day [31]. Dogs who received more social interactions with humans and other dogs and spent more time walking had shorter latencies to their first sleep bout and spent more time in quiet sleep than dogs that were less active during the day [31]. Furthermore, sleep patterns also correlate with age, such that older dogs tend to sleep more during the day and night, but their sleep is more fragmented (which is reflected in more, but shorter, sleep bouts), particularly throughout the day [32, 28]. Whilst previous observations often come from studies on very small samples (e.g. *N* = 1; [27]) and over very short periods of time (e.g. *N* = 3 hours; [31]), current research together highlights that sleep in dogs is sensitive to the environment, as in other human and non-human animals. Hence, individual differences in sleep patterns may reflect different degrees of adaptation and welfare, as argued by Ruckebush [8].

In this study, we investigated whether sleep could be used as a measure of welfare in shelter dogs. Since the assessment of welfare involves a number of behavioural, cognitive, and physiological proxies of a dog’s subjective wellbeing [33, 34], here a range of indicators were used, including judgement bias tests (see later), kennel staff observations, and day- and night-time behavioural observations from videos. Given how busy shelter environments are, and the close association between sleep and activity levels discussed above, a further aim of this study was to investigate how activity levels were related to sleep patterns, and whether dogs compensated for sleep deprivation by resting more. We hypothesised that dogs that experienced poor sleep quality would also show signs of compromised welfare on other indicators, and that, due to disturbances through husbandry procedures and increased stress, dogs would on average sleep less than previously reported. We also aimed to evaluate whether dogs that had a low percentage of time spent asleep during the night or day and highly fragmented sleep at night, slept and rested more during the day.

## Methods

### Subjects and housing

**Fig 1:**
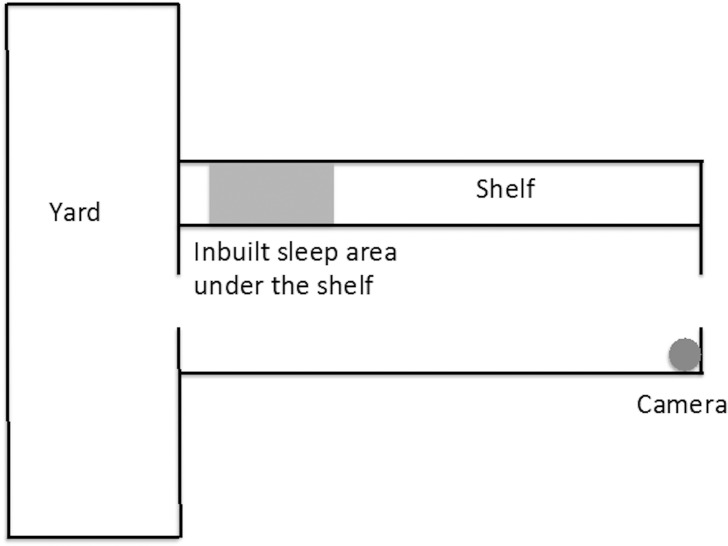
Layout of the kennel.

The study took place between March and October 2014. All dogs were kennelled in Battersea Dogs and Cats Home, Old Windsor. A total of 20 dogs were recruited for the study, but due to equipment failure, adoption, and unsuitable video quality, not all dogs could be included in all analyses (Table 1). Observations began after dogs had been in kennel for at least 10 days (range: 10 days -2 months), to allow for some initial adaptation to the environment. Dogs selected for this study were between 1–9 years old (mean age 3.9 ±2.5 S.D), comprising 30% females and 70% males, healthy and not receiving medication. A summary of dogs participating in this study is included in Table 1. Dogs were housed individually, three kennels apart from each other, minimising how much influence they had on each other’s behaviour. Each kennel was 3.8m x 2m with a concrete shelf to one side and an in-built bedding area underneath, where clean soft bedding was provided on daily basis (see Fig 1). During parts of the day, dogs had access to a 2 x 4 metre indoor run at the back of the kennel. Kennels were cleaned every morning between 08:00–10:00, during which time dogs were confined in the yard. All dogs in this study were fed twice a day, between 08:30–9:00 and at 15:30. Most dogs were fed a mixture of commercial complete dry food and tinned food. During most days, dogs were walked at least once and usually twice for 15–30 minutes or given access to a quiet room or kitchen area. Most dogs also experienced at least one 5 minute kennel session during which a volunteer or member of staff interacted with a dog in kennel.

**Table 1:** Summary of dogs participating in the study.

Dog	Age (months)	Sex	Breed (as recognised by the member of staff)	Acquisition	Elements of the study in which the dog participated or reason for exclusion
Alma^1^	41	f^2^	Staffordshire Bull Terrier cross	stray^4^	JBT^6^, NDO^7^, BO^8^
Benny	28	m^3^	Lurcher	stray	JBT, NDO, BO
Cyril	85	m	Mastiff cross	gift^5^	JBT, NDO, BO
Drake	29	m	American Bulldog	gift	JBT, NDO, BO
Erik	49	m	Lurcher	gift	JBT, NDO, BO
Flower	56	f	Lurcher	stray	JBT, NDO, BO
Glenn	81	m	German Shepherd cross	gift	JBT, NDO, BO
Hulk	52	m	Akita	gift	JBT, NDO, BO
Jake	105	m	German Shepherd	gift	JBT, NDO, BO
Kyla	21	f	Bull Mastiff	gift	JBT, NDO, BO
Loki	33	m	Akita x German Shepherd	gift	JBT, NDO, BO
Marley	57	m	Staffordshire Bull Terrier	gift	JBT, NDO, BO
Niko	31	m	American Bulldog	stray	NDO, BO (JBT not possible due to dog attending the rehoming meeting during the testing day)
Oonagh	23	f	Lurcher	stray	NDO, BO (JBT not possible due to dog being fearful of strangers)
Pam	30	f	Lurcher	stray	NDO, BO (JBT not possible due to dog attending the rehoming meeting during the testing day)
Quartz	43	m	Pearson Terrier	gift	JBT (NDO and BO not obtained as dog was rehomed during the observations)
River	78	f	Alaskan Malamute	gift	JBT (NDO and BO lost due to equipment failure)
Star	15	f	Labrador	stray	JBT (NDO and BO lost due to equipment failure)
Toby	22	m	Staffordshire Bull Terrier	gift	JBT (NDO and BO lost due to equipment failure)
Wilson	41	m	Staffordshire Bull Terrier cross	gift	JBT (NDO and BO lost due to equipment failure)

^1^All names were anonymised to protect the identity of the current owners

^2^female

^3^male

^4^stray: dog that arrived to the shelter after being found homeless

^5^gift: dog that was relinquished to the shelter by the owner

^6^JBT: judgement bias test

^7^NDO: night-time and daytime observations

^8^BO: 10 minute behaviour observations.

### Behaviour observations

#### Sleep and activity observations

**Fig 2:**
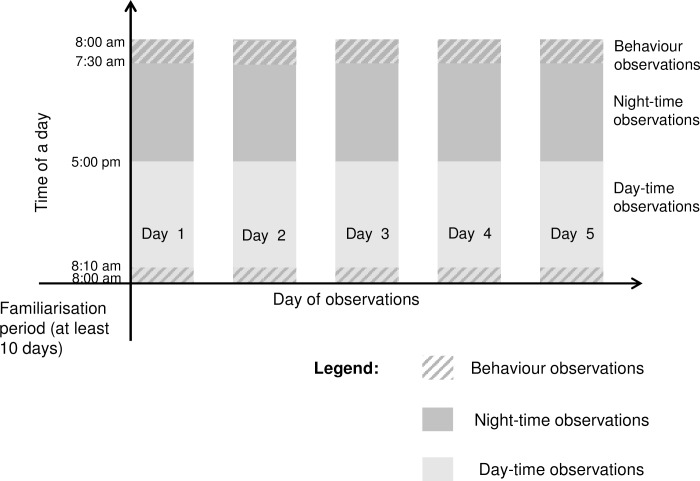
Observation schedule.

We used four CCTV cameras (Swann PRO- 735–700TVL with 4 Channel H.264 DVR recorder) with night vision capabilities for all observations. Each dog was observed every other day for nine days, resulting in five non-consecutive days of video per dog (see Fig 2). Observation periods were divided into night-time (17:00–08:00) and daytime (08:00–17:00), according to the shelter’s operational hours. Over each 24-hour period, the behaviour of the dogs was coded as active, resting, or sleeping, according to the ethogram in Table 2. Throughout the study, sleep encompassed REM and quiet sleep. However, as we did not use electroencephalography to quantify brain activity corresponding to sleep stages, sleep was defined operationally with reference to behaviours observed. For this reason, inaccuracies in measurements could have occurred. Continuous focal observations (to the nearest second) were used. A small percentage (0.2% for night-time and 5% for daytime) of observation data were lost or not suitable for analysis due to equipment failure, people in the kennel, or the dog being out of sight. To account for this, all observations were converted into proportions of the observation period before being analysed. The data from five days were averaged for each dog for statistical analyses. Definitions of sleep and activity metrics used in data analysis are presented in Table 3.

**Table 2:** Ethogram of sleep and activity measures.

Behaviour		Definition	Measure
Awake	Resting	The dog’s abdomen is touching the ground with its dorsal, caudal or lateral side whilst legs are extended forwards, curled close to the body or laid to one side. Eyes are open.	Duration (s)
	Active	Any other activity other than resting.	Duration (s)
Asleep	Asleep	As ‘Resting’ but eyes are closed for at least 2 minutes. Possible twitching of paws, ears, whiskers, tail or any of the above, eyes flickering and some vocalisations (muffed barks, whines, howling) could also be happening.	Duration (s)

**Table 3:** Definitions of individual sleep components used in analysis (following Zanghi *et al*., 2013 [28]).

Measure	Definition
Number of sleep bouts	Sum of all sleep bouts
Average duration of a sleep bout	Total duration of sleep during the phase divided by the number of sleep bouts
Percentage of a phase spent awake	Percentage of phase spent resting or being active
Latency to the first sleep bout	Time elapsed from staff leaving the kennels (17:00) to the first occurrence of a sleep bout
Latency to the activity onset	Time when the last episode of sleep ended. Negative number indicates time before staff arrival (before 08:00).

#### Behaviour-based indicators of welfare

An additional 10 minutes video coding between 07:30–08:10 from each of the five days per dog was conducted to code the occurrence of behaviours previously used as indicators of welfare, not included in the observation of activity levels described in section 2.2.1. This time period was selected because at that time most dogs were awake, there were no visitors and all dogs were still in the kennel. For this purpose an ethogram adapted from Titulaer *et al*. [3] was used (Table 4). Behaviours included the duration of: spinning, circling, pacing, vocalisations, and panting, which were converted to proportions of the observation periods and averaged between the observation days for each dog.

**Table 4:** Ethogram of behaviours used as welfare indicators (after Titulaer *et al*. [3]).

Behaviour		Definition	Measure
Repetitive	spinning	Dog moving vigorously (running) in a tight circle, possibly holding tail in mouth	Duration (s)
	circling	Dog is repetitively moving slowly (walking) in a circle	Duration (s)
	pacing	Dog walks or trots in a straight line, re-treading the same route repetitively.	Duration (s)
Panting	panting	Mouth open, tongue can be outside of mouth, quick and shallow breathing (inhalations–exhalations visible)	Duration (s)
Vocal	howling	Mouth continuous to be open in an ‘o’ shape, whilst the lower jaw moves up and down, the head is often directed upwards. Dog emits a continuous, tonal, high pitch vocalisation.	Duration (s)
	barking	Mouth opens and closes rapidly, lower jaw moves, dog emits short, noisy, loud vocalisation	Duration (s)

#### Staff observations

Trained kennel staff assessed each dog’s behaviour in the kennel as part of normal routines using predefined codes (not shown). As the codes could not be expressed on a scale, a single measure of proportions of days coded as ‘relaxed’ during the observation period was calculated for each dog.

#### Judgement bias tests

**Fig 3:**
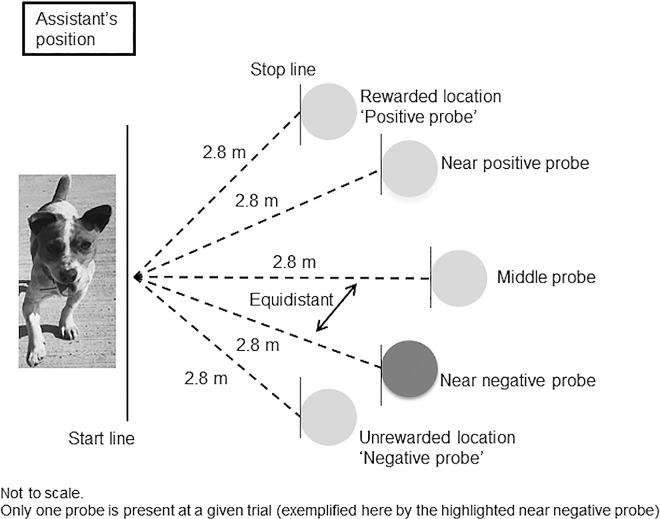
Judgement bias test set up.

A judgement bias was conducted following the procedure outlined in [35]. All tests were carried out between the first and third day of filming for each dog. After a 10 minute period of habituation with testers in the room, a researcher (SCOG) and a research assistant trained dogs to recognise that one food bowl (left or right, balanced between all dogs), contained food whilst the other bowl was empty. Commercially available treats (e.g. Schmackos) were used. Dogs received at least 15 training trials conducted in such a way that no location was repeated more than twice, and starting with two positive (rewarded) trials to encourage participation followed by two negative (non-rewarded) trials, with the order of remaining trials being random (Fig 3). When a dog had run six consecutive trials to the positive location faster than to the negative location, it was assumed that the task was learnt [35]. Latency to the bowl was recorded from the point a dog’s nose crossed a white line on the floor after being unclipped from a lead, until dipping their nose in a food bowl or stopping. The maximum time allowed per trial was 30 seconds. If the dog did not reach the food bowl in that time, the maximum time was recorded.

The test phase followed immediately after reaching criterion. Each dog was presented with a food bowl in three intermediate locations between the positive (P) and negative (N) bowl (labelled as near positive, NP, middle, M, and near negative, NN) (Fig 3). Each ‘probe’ was presented three times and followed by four training trials (two positive and two negative in a pseudo-random order, i.e. no location repeated more than twice in a sequence) to confirm previously trained responses to reference locations. After the final trial, a rewarded positive food bowl was placed in the unrewarded location to check if a dog’s performance in the trial was guided by olfactory cues or learning. The researcher (SCOG) was blinded to the location of rewarded bowl during training and trials and the research assistant who set the bowl was not visible to the dog during testing.

Dogs were considered to have a positive judgement bias if they ran to the middle probe at a similar pace they run to the positive (rewarded) probe, and a negative bias if they ran to the middle probe at a pace similar to which they ran to the negative (unrewarded) bowl. Similar pace was defined as within 15% of the pace to the rewarded or unrewarded bowl. To account for size differences, which could affect the running speed of a dog, all latencies were adjusted before regression analysis using the approach taken in Mendl *et al*. [35].

### Statistical analysis

All data were analysed in R version 3.1.3. All data were explored graphically and normality was assessed with Shapiro-Wilk tests. Data were either transformed to meet the assumption of normally distributed residuals in linear regressions or non-parametric statistics were used. Percentage of night spent asleep and the number of sleep bouts during the night (i.e. sleep fragmentation) were collinear. Therefore, the percentage of time spent asleep during the night was selected as the primary measure of sleep for ease of comparison with previous publications (e.g. [28]). The percentage of time spent asleep during the day and the percentage of time spent resting during the day were selected as dependent variables, because previous studies (e.g. [23]) suggest that changes in resting may indicate lower welfare.

#### Effect of age on sleep pattern

Correlations between age and sleep or activity measures were explored using Pearson’s Product Moment Correlation.

#### Quality of sleep at night and activity during the day

To investigate if activity during wakefulness predicts sleep measures at night, and if dogs who lost sleep at night compensate by sleeping more during the day, a multiple linear regression model was used. The percentage of time spent asleep during the night was the dependent variable, and the percentage of time spent asleep during the day and percentage of time spent resting during the day were independent variables.

#### Judgement bias

The effect of bowl position on the unadjusted latency to approach in judgement bias testing was analysed using a Friedman test. Post-hoc pairwise Wilcoxon tests with Bonferroni corrections for multiple testing were used to see if latency towards each probe differs significantly from those at all other locations.

#### Existing measures of welfare and sleep

To explore the relationship between existing behavioural indicators of welfare and sleep measures, three multiple regression models were constructed: The dependent variables were: 1) the percentage of time spent asleep during the night; 2) the percentage of time spent asleep during the day; and 3) the percentage of time spent resting during the day respectively. In each model, the independent variables were: 1) repetitive behaviour, 2) vocal behaviour, 3) latency to approach the middle probe and 4) the percentage of time coded as ‘relaxed’. Statistical significance was set to α < 0.05. Panting was dropped from the analysis as only a few dogs showed it.

### Ethical approval

This study was carried out in accordance with the University of Lincoln ethical guidelines and with Battersea Dogs and Cats Home permission. The research protocol was approved by the University of Lincoln Research Ethics Committee (no. COSREC97).

## Results

Of the 20 dogs recruited for the study, 100% (n = 20) had staff recordings, 81% (n = 17) participated in the judgement bias test, 71% (n = 15) were video recorded during the day and night, and data on all variables were available for 57% (n = 12) of dogs. 10% of all recordings were randomly selected and coded for the second time approximately four months after the original coding. The intra-rater agreement reached 92%.

### Characteristics of sleep pattern

A total of 1800 hours of observation were analysed. Descriptive statistics for measures of sleep and activity were calculated for each dog (Table 5 and data in S1 Table). The percentage of time spent asleep during the night showed a strong negative correlation with sleep fragmentation, measured by the number of sleep bouts during the night (Pearson’s *r* = -0.80, n = 15, p <0.01). On average, dogs in this study spent 660.39 minutes asleep over 24-hour period (44.9% ± S.E. 11.6). The average activity onset time was 77.6 (±S.E. 20.1) minutes before the arrival of staff (corresponding to 6:43 a.m.). Dogs entered sleep bouts on average 16.9 (±S.E. 4.7) minutes after staff departure (corresponding to 17:16 p.m.).

**Table 5:** Activity during the day and night. See table in S1 Table for the individual differences between dogs.

Variable	Average ± S.E. Day time	Average ± S.E. Night time
Phase duration (min)	540	720
Percentage of phase spent asleep (%)	2.60 ± 0.47	71.62 ± 1.59
Number of sleep bouts (n)	1.60 ± 0.34	32.81 ± 3.16
Average duration of a sleep bout	23.59± 2.07	24.52 ± 3.12
Total of a phase spent resting (%)	23.59± 2.07	20.54 ± 1.49
Total of a phase spent active (%)	73.80± 2.13	6.99 ± 0.69
Latency to the first sleep bout (min)	N/A	16.94 ± 2.57

### Age and sleep

There was no statistically significant effect of age on any of the sleep and activity measures during the day or night.

### Activity during daytime and sleep at night

The multiple regression model regressing the percentage of time spent asleep during the night on the percentage of time spent asleep during the day and the percentage of the day spent resting was not significant (*F*_2,8_ = 1.18, *R*^2^ = 0.23, p>0.05, n = 15).

### Sleep and judgement bias

**Fig 4:**
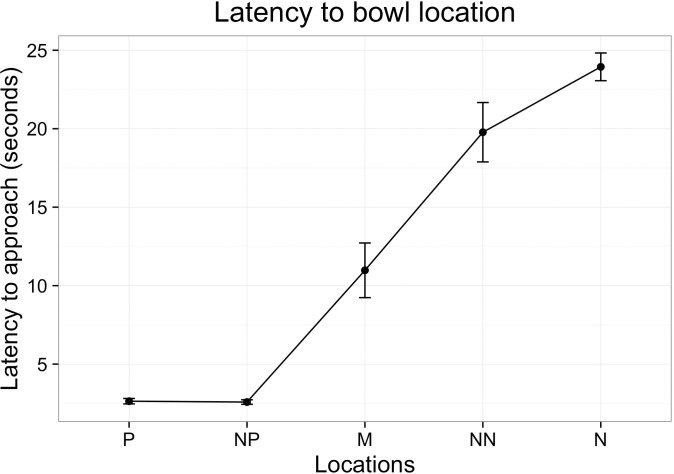
Latency to approach probe (±S.E.). P–positive probe, NP- near positive probe, M- middle probe, NN- near negative probe, N- negative probe.

Bowl position had a significant effect on latency to approach the bowl (*X*^2^ = 58.4, d.f. = 4, n = 17, p <0.0001). With the exception of P vs. NP and N vs. NN probes (p> 0.05, Fig 4 and Table 6), all locations were significantly different from each other (t = -4.81 for NP vs M probes and t = 3.42 for M vs. NN probes, p<0.001). At the end of experiment, the bowl from the positive location was put in the negative location to test if dogs were guided by learning or odour cues. The average latency to reach this bowl was 16.2 s (S.D. 3.9) vs. 2.6s (S.D. 0.64) for positive location.

**Table 6:** Latency to approach test probes.

Latency to approach		
Probe location	Average ± S.E. (s)	Adjusted score ± S.E
Positive	2.64 ± 0.64	N/A
Near positive	2.58 ± 0.63	-0.35 ± -0.09
Middle	10.98 ± 2.66	38.11 ± 9.24
Near negative	19.78 ± 4.80	82.74 ± 20.07
Negative	23.94 ± 5.81	N/A

### Associations between sleep, rest and behavioural indicators of welfare

**Fig 5:**
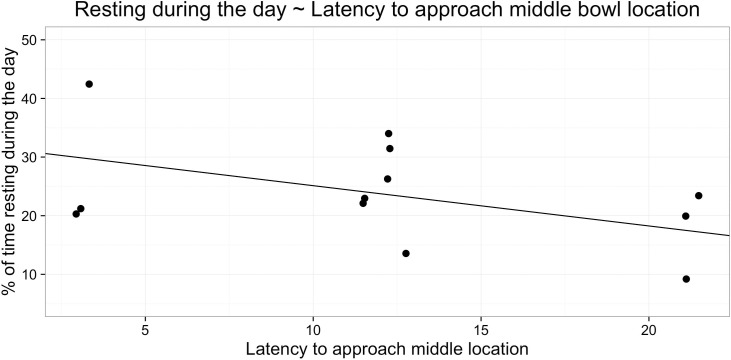
Relation between resting during the day and latency to approach middle probe.

**Fig 6:**
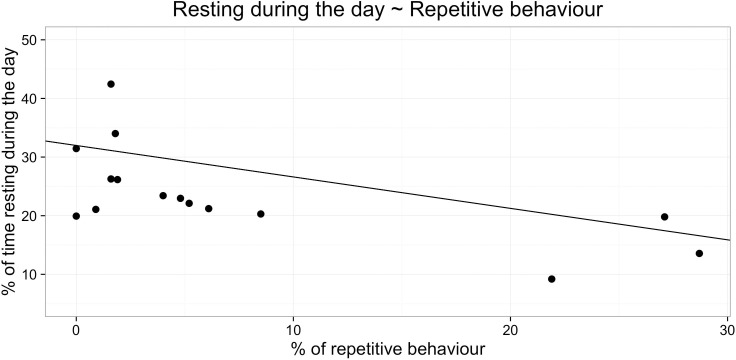
Relation between resting during the day and percentage of time showing repetitive behaviour.

The linear regression model predicting percentage of time during night or day spent asleep from behavioural indicators of welfare was not significant (day time sleep: *F*_4,7_ = 0.48, *R*^2^ = 0.21, p > 0.05, n = 12; night time sleep: *F*_4,7_ = 0.37, *R*^2^ = 0.18, p > 0.05, n = 12). However, the multiple regression model predicting resting behaviour during the day was significant and explained 80% of variation in resting behaviour (*F*_4,7_ = 7.14, *R*^2^ = 0.80, p<0.05, n = 12). When resting behaviour was modelled, it was found that latency to approach the ambiguous middle probe *β* = -0.17, t = -2.93, p < 0.05, Fig 5 and repetitive behaviour *β* = -0.58, t = -3.11, p < 0.05, Fig 6) were significant predictors of time spent resting during the day, whereas the percentage of time coded as ‘relaxed’ was approaching significance (*β* = 0.13, t = 2.36, p = 0.0506).

## Discussion

This study is the first to investigate whether sleep can be used as a measure of welfare in shelter dogs. It aimed to quantify the sleep pattern of shelter dogs, analyse the relationship between activity during the day and sleep patterns, and compare sleep patterns to other behavioural and cognitive indicators of welfare.

### Sleep patterns of shelter dogs

Overall, dogs slept for 660.4 minutes over the 24-hour period (44.9%±11.6): 71.8% of the night-time (range: 83.30± 8%–61.19± 1.11%) and 2.8% (range: 6.25± 1.69%–0) of the daytime. Dogs in this population slept less over 24-hours than previously reported. For example, Zanghi *et al*. [28] reported 733 minutes of sleep over 24-hours (50.9%). Other studies reported between 31% [28] and 26% [32] of sleep during the daytime. When corrected for observed time, shelter dogs slept on average for 71.6% of the night, which is exactly the same as the amount reported by Zanghi *et al*. [29]. However, dogs slept substantially less during the day (14.3% in [29]). Therefore, the main difference between the current study and previous publications is the absolute value of the proportion of day spent asleep and the distribution of sleep over 24-hours. Previous studies reported frequent sleep bouts distributed evenly throughout the day [27, 28, 29] or clustered around 12:00 and 16:00. Here, dogs slept primarily after 17:00 and throughout the night, when the shelter was closed. It is likely that shelter dogs experience a ceiling effect in respect to how much sleep they can get during the day due to the busy shelter routine. As sleep is homeostatically regulated and motivation to sleep increases with time since the previous sleep bout [36], dogs may adapt to this ceiling effect by sleeping more throughout the night. For instance, Kis *et al*. [31] found that sleep depriving dogs for a short period throughout the day resulted in a shorter latency to the first sleep bout, a marginally higher percentage of time spent asleep, and longer slow-wave sleep. In support of this, a slight decrease in the latency to the first sleep bout in the study reported here (18 minutes after the onset of night-time compared with 28 minutes in Zanghi *et al*. [29]) suggests that dogs were strongly motivated to sleep but were possibly unable to fulfil this need during the day.

On average, dogs had 32 sleep bouts/night (range: 18.5±0.33–63.0±2.98), which is much lower than previous reports (e.g. 60.3 in Takeuchi & Harada [32]; see also Kis *et al* [31]). Prolonged wakefulness is compensated for by an intensification of slow-wave brain activity during quiet sleep and, as a result, a decreased number of spontaneous awakenings during the sleep phase [37]. Although inaccuracies of direct observations could underestimate the degree of sleep fragmentation, the data here suggest that shelter dogs had less fragmented sleep than previously reported, which could be a result of altered sleep architecture in response to sleep deprivation during the day. Previous research shows changes in REM sleep patterns following both chronic [10] and acute stress [11]. In this study, it was not possible to code REM sleep accurately, therefore this measure was dropped. Using alternative methods of observations, such as accelerometers [29], could allow the exploration of whether changes in REM patterns occur in shelter dogs.

No significant relationship between age and sleep pattern was detected, although the statistical trends observed were in the same direction as previous research: older dogs tended to spent greater percentage of time asleep during the night, had slightly more sleep bouts during the night and spent a lower percentage of day asleep [29, 32]. The differences in this study are likely not to have been as striking as previously reported due to the younger age of dogs participating (maximum age of 8.9 here vs. average age of 16 in Takeuchi & Harada [32]).

### Sleep, activity levels and measures of welfare

A main objective of this study was to explore the relationship between sleep behaviour at night and sleep and activity during the day. One of the hypotheses considered under this study was that dogs who spend a smaller proportion of the time during the night asleep either compensate by sleeping or resting more during the day or are unable to sleep or rest efficiently throughout the day, due to being less well adapted to the environment. We found that the percentage of time spent asleep during the night was not significantly predicted by time spent resting during the day, nor the proportion of time spent asleep during the day. As discussed above, it is possible that the shelter environment imposes a ceiling effect on the amount of time that can be spent asleep or resting during the day, which complicates the relationship between daytime activity and night time sleep. Moreover, research by Kis *et al*. [31] suggests that variation in sleep patterns (e.g. latency until first bout) are sensitive to even short periods of sleep deprivation/activity. The data reported here were aggregated over several days to for each dog, which could have obscured the day-to-day variation in sleep–activity rhythms. What is more, day time activity could have affected the internal architecture of night time sleep (e.g. latency to the first REM sleep bout or duration of individual REM sleep bouts [10]), rather than the overall quantity of sleep. Coupled with our small sample size, it is possible that our analysis was not sensitive enough to detect the relationship between night time sleep and activities during the day. Future research should investigate how activity time budgets between sleep and resting may change throughout the day, rather than only using mean values.

Contrary to our hypothesis, the percentage of time spent asleep during the night was not predicted by behavioural indicators of welfare, including the judgement bias test. It is possible that the relationship between sleep and behavioural indicators is non-linear: sleep that is above or below the optimal amount of sleep correlates with a negative mood and poor welfare in dogs. However, the reverse is also plausible: changes in sleep could be a result of negative mood in dogs. Studies on humans suggest that the relationship between sleep and health as well as sleep and wellbeing follow a non-linear pattern–a convex down parabola, with health and wellbeing outcomes being worse for people who sleep below and above average [38, 39]. Optimism and the amount of sleep at night follow the same pattern among children: children on both ends of the continuum of time spent asleep at night are less optimistic than those in the middle [40]. This sleep pattern is also typical in patients suffering from depression, where both excessive sleep and insomnia are common symptoms [41]. It is also plausible that changes in internal sleep architecture reflect welfare more than changes in the overall quantity of sleep.

Nonetheless, dogs that rested more showed a more positive judgement bias, less repetitive behaviour and received a higher percentage of ‘relaxed’ codes by the kennel staff. Therefore, the data suggest that greater resting behaviour during the daytime is a clearer indicator of good welfare in shelter dogs than a greater proportion of sleep during the night. Why might this be the case? Here, repetitive behaviours were only observed in the 10 minute morning slots, when dogs could be reacting to audible background noise of staff arriving to the shelter. Denham *et al*. (2014) [42] demonstrated that most repetitive behaviour in kennel environments is in response to specific stimuli. The authors [42] showed that whilst repetitive behaviour could be a sign of poor welfare, the motivation underlying repetitive behaviour is complex and could be a result of differential arousal levels in response to a stimulus. Higher activity levels in kennel is a natural response to a more stimulating environment [43], so, differences in resting behaviour among dogs housed in the same environment could be explained by how easily a dog is aroused by external stimuli. Within-individual differences in resting behaviour could reflect day-to-day changes in the environment and average between-individual differences could indicate degrees of adaptation to the shelter environment. Previous research illustrates that dogs more habituated to kennel environments possess lower cortisol:creatinine levels and have reduced startle responsiveness compared to dogs less habituated [43]. Stress hormones, like cortisol, have been hypothesised to prepare for a stressor by increasing responsiveness to the external stimuli [44, 45]. Given the limits to the amount of sleep feasible during the day in shelter environments, the results here suggest that those dogs that rest more during the day may be better adapted and have higher welfare.

However, resting should still be interpreted with caution, as it could also occur due to apathy or learned helplessness, or be a product of less stimulating environments. For instance, Meagher *et al*. [23] observed that mink living in unenriched cages spent more time resting (see also: [46, 24]). An alternative explanation of the results of the judgement bias test also needs to be considered. The intended ‘neutral’ testing conditions could actually be rewarding for shelter dogs. Being taken out of the kennel into a novel environment where the test was conducted, together with receiving human contact and the short walk from the kennel to the test location, could have had a positive impact on the dogs’ mood [47]. Dogs who are experiencing poorer welfare could benefit more than dogs experiencing better welfare, leading to those dogs with poorer welfare seemingly having more positive mood. More generally, it should be noted that the predictive value of behaviour-based welfare indicators has recently been called into question in comparison to physiological measures [48]. It is therefore necessary that sleep, resting and physiological data from dogs be explored thoroughly to elucidate the value of sleep as a measure of welfare.

### Recommendations

Introduction of ‘quiet time’ when access of visitors is restricted and noise levels kept to minimum could encourage more sleeping and resting behaviour during the day and have a positive impact on dog welfare.

## Conclusion

This study found that dogs in shelters sleep substantially less during the day than previously reported, likely due to the busy shelter environment. Although our results cannot demonstrate a causal relationship, it is possible that dogs adapted their sleep routine to this environment by sleeping earlier and reducing sleep fragmentation throughout the night. Non-significant relationships between time spent asleep, judgement bias, and behaviour-based measures of welfare suggest sleep may not be useful as a welfare indicator in shelter dogs. However, non-linear interactions between sleep and wellbeing in humans, coupled with the low sample size here, means that this study was possibly not able to detect more complex relationships between these variables in dogs. Increased resting during the day emerged as a more robust sign of welfare. Future research should help to elucidate the casual relationships between sleeping and resting and positive welfare to help answer if better welfare is achieved by resting and sleeping more during the day, or whether resting and sleeping is a consequence of better welfare.

## Supporting Information

S1 Table Individual differences in sleep and activity measures. A table with summaries of sleep, activity and resting measures for each dog.
(DOCX)

S2 Table Judgement bias test results for all dogs. (XLSX)

S3 Table Percentage of time coded as ‘relaxed’ by the member of staff. (XLSX)

S4 Table Sleep and activity measures for each dog (XLSX)

S5 Table Averages of welfare data. Averaged scores for all welfare measurements for each dog (i.e. repetitive behaviour, vocal behaviour etc.).
(XLSX)
