# Supplementary material for: Can Sleep and Resting Behaviours Be Used as Indicators of Welfare in Shelter Dogs (Canis lupus familiaris)?
Source: PLoS One. 2016 Oct 12;11(10):e0163620. doi: 10.1371/journal.pone.0163620 (PMC5061428; doi:10.1371/journal.pone.0163620)
Supplement: S1 Table — A table with summaries of sleep, activity and resting measures for each dog. (DOCX) [file pone.0163620.s001.docx]

**S1 Table. Individual differences in sleep and activity measures.**

| Dog | Proportion of daytime^1^ spent asleep ± S.E. (in minutes) | Number of sleep bouts during daytime ± S.E. | Proportion of daytime spent resting or being active ± S.E. | Proportion of night-time^2^ spent asleep ± S.E. (in minutes) | Number of sleep bouts during night-time ± S.E. (in minutes) | Proportion of night-time spent resting or being active ± S.E. | Latency to the first sleep bout (in minutes) ± S.E. |
| --- | --- | --- | --- | --- | --- | --- | --- |
| Benny | 0.40 ± 0.15 | 0.6 ± 0.14 | 9.20±0.78,90.41±0.80 | 66.18 ± 0.44 | 44.4±2.09 | 23.89 ±1.06, 10.52± 1.65 | 13.71±0.002 |
| Hulk | 1.79 ± 0.75 | 0.6 ± 0.23 | 34.00± 3.33, 64.21± 2.93 | 83.30 ±8.0 | 25.2±2.28 | 13.23±6.94, 2.97±2.08 | 16.99±0.003 |
| Loki | 2.17 ± 0.35 | 1.6 ± 0.35 | 42.44 ± 1.42, 55.38 ± 0.97 | 64.13±0.91 | 42.8±1.15 | 27.33±0.77, 7.17± 1.23 | 28.51±0.003 |
| Jake | 0 | 0 | 21.20 ± 5.18, 78.80 ± 5.18 | 65.21±0.49 | 63±2.98 | 26.06±2.61, 6.06±0.86 | 12.84±0.002 |
| Kyla | 6.25 ± 1.69 | 4.4±1.0 | 26.27 ± 2.0, 67.48 ± 3.1 | 70.04±1.16 | 47.4±1.55 | 21.61±0.43, 6.99±0.8 | 9.43±0.001 |
| Alma | 4.40 ± 0.94 | 1.6 ± 0.39 | 13.56 ± 1.63, 82.04 ± 1.14 | 79.43±1.44 | 21.2±4.34 | 13.80±1.58  6.73±0.27 | 9.23±0.001 |
| Niko | 2.87 ± 0.62 | 2.5 ± 0.68 | 21.08 ± 1.47, 76.05 ± 1.41 | 71.42±0.34 | 18.5±0.33 | 10.89±0.83, 13.39±1.5 | 16.03±0.003 |
| Marley | 3.13 ± 0.63 | 2.0 ± 0.54 | 22.96 ± 1.49, 73.92 ± 0.82 | 68.95±0.15 | 35.0±2.92 | 26.24±2.16  4.57± 2.92 | 40.55±0.003 |
| Cyril | 3.64 ± 0.91 | 1.8 ± 0.56 | 20.29 ± 3.29, 76.07 ± 2.73 | 78.83±1.71 | 25.4±3.01 | 13.38±1.85, 6.80±0.87 | 15.2±0.003 |
| Drake | 3.56 ± 0.74 | 4 ± 0.73 | 22.11 ± 1.68, 74.33 ± 1.24 | 71.38±0.39 | 36±1.28 | 23.45±0.69, 3.91±0.21 | 6.26±0.0001 |
| Erik | 5.32 ± 0.43 | 2.6 ± 0.35 | 23.41 ± 1.23, 71.27 ± 6.25 | 73.32± 1.49 | 30.6±1.30 | 18.85±1.55, 7.61±0.92 | 26.17±0.002 |
| Glenn | 1.91 ± 0.81 | 0.8 ± 0.34 | 31.45 ± 2.01, 66.64 ± 2.5 | 77.60±1.18 | 20.0±1.30 | 16.26±1.16, 6.14±0.11 | 11.40 ±0.002 |
| Flower | 1.61 ± 0.64 | 0.4 ± 0.14 | 19.93 ± 0.95,78.45 ± 0.69 | 72.28±0.76 | 24.4±0.87 | 21.11±0.48, 6.60±0.29 | 16.13±0.003 |
| Oonagh | 1.66 ± 0.85 | 0.75 ± 0.39 | 19.80±1.77, 78.55±1.63 | 61.19±1.11 | 28.25±0.49 | 28.38±0.86, 10.08±0.93 | 24.0±0.003 |
| Pam | 0.39 ± 0.17 | 0.33 ± 0.15 | 26.16±1.75, 73.46±1.90 | 70.97±0.13 | 30.02±1.18 | 23.70±0.26, 5.25±0.14 | 34.89± 0.003 |
